# Anatomy and Histology of the Mouth and Teeth

**Published:** 1910-03-15

**Authors:** 


Anatomy and Histology of the Mouth and Teeth. This is the third edition of Broomell's well known work, in the revision of which Philipp.Fischelis, M.D., of the Medical Faculty of the Medico-Chirurgical College of Philadelphia, has materially assisted. The material and its formal presentation has ondergone little change from the second edition. About twenty new illustrations are added, some changes in nomenclature to make it conform to scientific anatomic nomenclature, and the addition of new matter on the embryology and histogenesis of the cell, with especial reference to formation of the tissues and organs of the mouth. No work on dental anatomy gives more satisfactorily the anatomy of the teeth and gum structures. The illustrations are so well done as to in some cases render the text superfluous. The size of the book seems to have been decreased, but thisis the result of smaller type and thinner paper. It is however a beautifully made book and a welcome addition to our resources.

